# Preparation of Novel Banana-Shaped Triple Helical Liquid Crystals by Metal Coordination

**DOI:** 10.3390/ma2010146

**Published:** 2009-03-13

**Authors:** Parvez Iqbal, Manickam Mayanditheuar, Laura J. Childs, Michael J. Hannon, Neil Spencer, Peter R. Ashton, Jon A. Preece

**Affiliations:** 1 School of Chemistry, The University of Birmingham, Birmingham, Edgbaston, United Kingdom, B15 2TT; E-Mails: p.iqbal@bham.ac.uk (P.I.); m.j.hannon@bham.ac.uk (M.H.); n.spencer@bham.ac.uk (N.S.); p.r.ashton@bham.ac.uk (P.A.); j.a.preece@bham.ac.uk (J.P.); 2 Department of Chemistry, University of Warwick, Coventry, United Kingdom, CV4 7AL; E-Mail: laurachilds10@yahoo.co.uk

**Keywords:** Metallomesogens, banana, liquid crystals, triple helical, mesogenic.

## Abstract

The synthesis of a series of banana-shaped structures has been carried out, in which the bend unit is formed by a 4,4’-methylenedianiline or 3,3’-methylenedianiline core bearing two symmetric pyridylimine linkages to di- and tri- alkoxyphenylester moieties on the side arms. The molecules, in addition to providing an elongated aromatic central core associated with liquid crystal (LC) molecules, also provide binding sites for metals. The methylenedianiline spacer incorporates phenylene groups that sterically prevent the two binding sites from co-ordinating to a single metal centre and the central methylene unit introduces enhanced flexibility into the ligand backbone. Furthermore, complexes have been formed by the co-ordination between 3, 3’-methylenedianiline containing ligands and Cu (I) ions [Cu_2_(**3a-c**)_2_][PF_6_]_2_. Electrospray Mass Spectrometry (ESMS) and Fast Atom Bombardment Mass Spectrometry (FABMS) showed the formation of dimeric species; [Cu (L)_2_][PF_6_]_2_. Finally, thermal analysis of the ligands (**1a-d, 2a-d, 3a-c** and **4a-d**) and Cu complexes [Cu_2_(**3a-c**)_2_][PF_6_]_2_ has been carried out in order to investigate the phase properties of these materials. None of the banana-shaped ligands and the metal complexes [Cu_2_(**3a-c**)_2_][PF_6_]_2_ showed any mesophases.

## 1. Introduction

Although classical thermotropic liquid crystals (LCs) are commonly composed of rod-like molecules, many liquid crystals exhibit unconventional molecular structures. An example of such a molecular architecture is a banana-shaped one, with a bend in the middle of the mesogenic part [1,2,3,4]. Niori *et al.* [5] first observed ferroelectricity in a smectic phase which was produced from achiral banana-shaped molecules. Since then, several other research groups have [6] reported banana-shaped molecules that exhibited ferroelectric properties in the mesophase. The ferroelectricity is attributed to the polar packing of molecules with C_2v_ symmetry where the molecules are packed in the same direction. A great many analogous banana-shaped structures have been prepared, some with lateral substituents in the central aromatic ring, which exhibit the wide range of ‘banana phases’ commonly designated B_1_ through to B_7_ [7]. The existence of such mesophases depends on the length of the rigid core, as well as on the magnitude of the bend angle and its position. The structure and flexibility of the angled segment are also important [8]. 

Dating back to the 1980s there has been immense interest in metal-containing mesogens, and subsequently a novel subfield in liquid crystals has emerged, known as metallomesogens. One of the factors contributing to this growth of interest has been due to the presence of the metal atom causing significant change in the magnetic, electric and optical properties of liquid crystals [9,10,11]. Many liquid crystals calamites based on rare earth element complexes [9] have exhibited enhance magnetic susceptibility among liquid crystals, for example Dy and Tb complexes with Schiff’s bases and β-aminovinyl ketones demonstrate high anisotropies of magnetic susceptibility [9]. At present, the molecular motifs of the known metallomesogens are based on the classical rod-like [12,13,14,15] and disc-shaped mesogens [16]. One of the modern pioneers in this field is Bruce *et al.*, who has demonstrated [12,13,14,15] that by a suitable choice of anisotropic ligand, it is possible to form rod-like liquid crystals based on the metals with octahedral coordination geometry [12,13]. Strong chelating moieties such as 2,2-bipyridine [12] and imine [13] moieties have been utilised as the metal chelating units in these metallomesogens. Also, Ziessel *et al.*, have prepared several ligands based on 2,2’-bipyridine and these ligands assembled into a dinuclear double-helicate complex with copper that exhibits columnar mesophase [17].

**Scheme 1:**
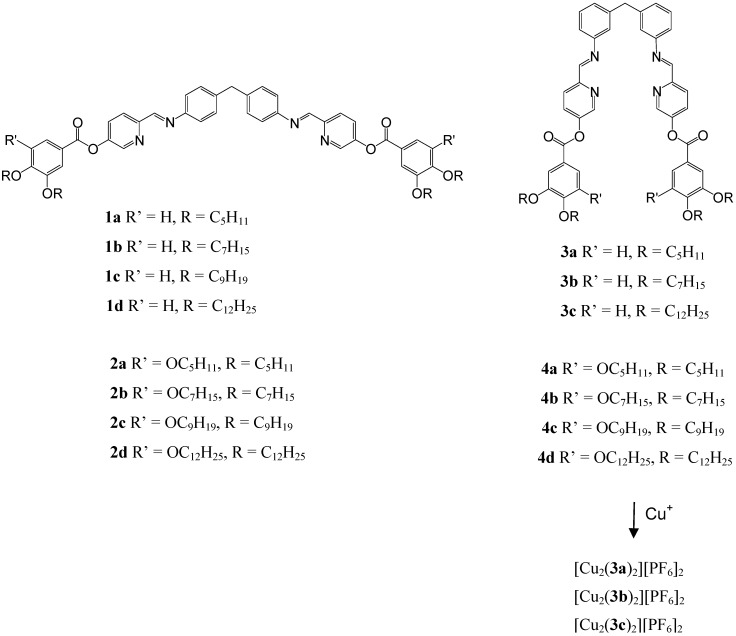
Desired banana-shaped molecules and the preparation of Cu complexes.

Hannon *et al.* [18,19] have shown that bispyridylimine ligands can form double or triple stranded helicate structures. The triple helicate arises through the entwining of three bispyridylimine ligand strands around two metal cations, while the double helicates arise with tetrahedral metal ions. Herein, two novel series of banana-shaped molecules have been prepared that incorporate metal binding units (Scheme 1). The binding units contain 4,4’-methylenedianiline and 3,3’-methylenedianiline moieties as the central units which are linked to pyridine-2-carbaldehyde, affording imine structures. It is envisaged from the chemistry of the unsubstituted analogues (X-ray structures of their complexes are known [18,19,20]) that the helicates formed from the 4,4’-methylenedianiline core will form gross linear structures [18,21] and hence chemical modification at the terminal of this helicate with alkyl chains will result in classical rod-like mesogenic structures. Whereas, the helicates formed from the 3,3’-methylenedianiline which is structurally constrained will develop a kink at the centre of the helicate [20] and thus, with alkyl chains at the terminal of the helicate will form novel banana mesogenic structures [Cu_2_(**3a-c**)_2_][PF_6_]_2_. The thermal properties of these ligands (**1a-d**, **2a-d**, **3a-c** and **4a-d**) and complexes [Cu_2_(**3a-c**)_2_][PF_6_]_2_ have been investigated. 

## 2. Results and Discussion

### 2.1. Design 

The exact relationship between angled molecular structures and liquid crystalline properties is still not very clear to date, but empirical rules about molecular design [22,23,24,25,26,27] have shown that this new class of banana mesogen is extremely sensitive to structural modification regarding the attainment of mesophases. For example, flexibility [22,28], bend angle [7,28], nature of the central core [22,29,30,31], length [26,31] and nature [32] of the terminal chains, substituents both on the central [7,26], and on the side arms [33], dipoles [26], and direction of linking groups [34] are among the main variants contributing to the capability of a material to exhibit liquid crystalline properties.

Here a synthetic scheme has been devised that allows the incorporation of 4,4’-methylenedianiline and 3,3’-methylenedianiline units as the central core of the molecular architecture, which as well as providing the bent core for the banana-shaped molecules, provide pyridylimine and methylene dianiline units for binding to metal ions.

### 2.2. Synthesis

**Scheme 2:**
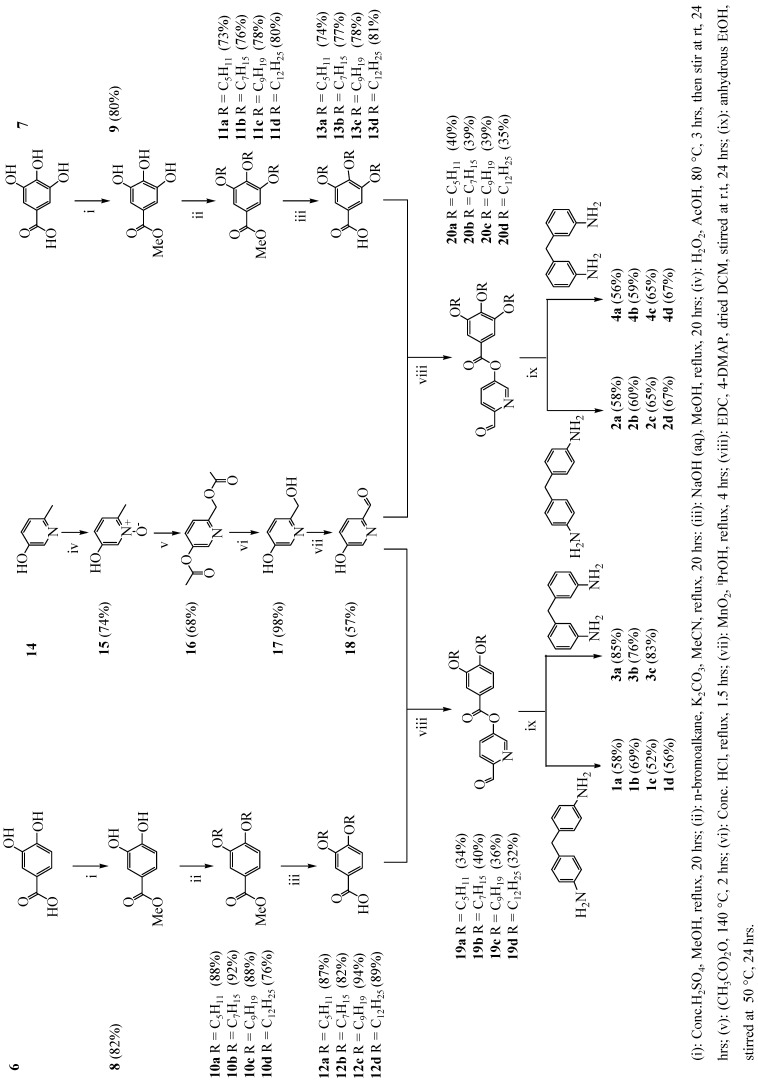
Synthesis of the desired banana-shaped molecules

The syntheses of the novel banana-shaped compounds (**1a-d**, **2a-d**, **3a-c** and **4a-d**) were achieved via a series of reactions as illustrated in Scheme 2. The commercially available di- and trihydroxy benzoic acids **6** and **7** were converted to their respective methyl esters in the presence of a catalytic amount of concentrated H_2_SO_4_ in MeOH to afford compounds **8** and **9.** Alkylation of compounds **8** and **9** was achieved under basic conditions (K_2_CO_3_) with *n*-bromopentane, *n*-bromoheptane, *n*-bromo-nonane and *n*-bromododecane, respectively affording compounds **10a-d** and **11a-d** [13]. The esters **10a-d** and **11a-d** were hydrolysed to the corresponding acids **12a-d** and **13a-d** under basic conditions (NaOH) [13]. The commercially available 5-hydroxy-2-methylpyridine (**14**) was directly oxidised with H_2_O_2_ to afford the N-oxide **15**. The N-oxide was subsequently heated in acetic anhydride to afford the acetate **16**. Under acidic conditions (concentrated HCl) compound **16** was deprotected to afford the alcohol **17**. Selective oxidation of the alcohol moiety was achieved in the presence of a mild oxidising agent, MnO_2_ [35], to afford compound **18**. Compound **18** was then coupled with the acids **12a-d** and **13a-d** through dicyclohexylcarbodiimide (DCC) [13] coupling to afford the respective aledehydes **19a-d** and **20a-d**. The target imine compounds **1a-d**, **2a-d**, **3a-c** and **4a-d** were obtained via stirring under N_2_ atmosphere at room temperature between the respective aldehydes **19a-d** and **20a-d** and methylene dianilines [36]. Finally, the complexes [Cu_2_(**3a-c**)_2_][PF_6_]_2_ were formed by coordination of molecules **3a-c** to Cu (I) ions at room temperature in *iso*-PrOH [18,21].

### 2.3. Thermal Properties of Bent-shaped Ligands

**Figure 1:**
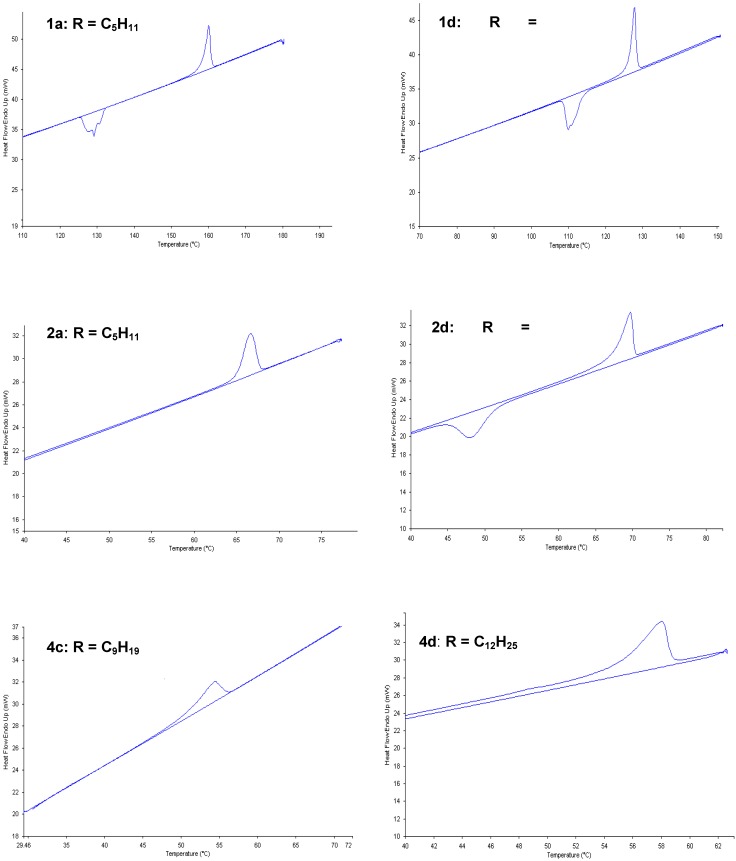
DSC traces for compounds **1a**, **1d**, **2a**, **2d**, **4c and 4d** (second heating cycle at a rate of 5 °C/min).

Thermal analysis was carried out on compounds **1a-d, 2a-d, 3a-c** and **4a-d** using variable temperature optical polarised microscope (OPM) between crossed polarisers, and differential scanning calorimetry (DSC). OPM revealed that upon heating from solid to the liquid phase, no birefringent liquids were formed, and the materials passed straight to the isotropic liquid phase. On cooling the I→K transition was observed at slightly lower temperatures, confirming the lack of any mesophase formation. However, for a few of the compounds (**2a, 4c-d**) the I→K transition was not observed. This result suggests that these compounds decomposed through the K→I transition. As expected, for all the **1a-d**, **2a**, **2d**, **4c and 4d** derivatives, the K→I transition decreased with increasing the length of the carbon chain. The DSC traces are illustrated in Figure 1 and the melting points are listed in Table 1**.**

**Table 1:** Transition temperatures (onset values) (°C) and enthalpy values ΔH (kJ/mol) recorded on second heating by DSC (10 °C/min) for compounds **1a-d, 2a, 2d, 4c** and **4d.**

Entry	K→I	ΔH
**1a**	158	81.3
**1b**	136	64.3
**1c**	129	40.0
**1d**	126	81.0
**2a**	67	43.7
**2d**	51	115.8
**4c**	51	60.8
**4d**	55	85.4

### 2.4. Metal Complexes

Cu complexes [Cu_2_(**3a-c**)_2_][PF_6_]_2_ were isolated as red precipitates and through UV/vis spectroscopy λ_max_ are determined as 495, 485 and 480 nm, respectively, which are characteristic for Cu (I) ions in a bis-pyridylimine environment [18,20,21]. The masses obtained for complexes [Cu_2_(**3a**)_2_][PF_6_]_2_ and [Cu_2_(**3b**)_2_][PF_6_]_2_ from ES and FAB mass spectrometry, show that the species formed are dinuclear double stranded arrays in the form of [Cu_2_(**3a-c**)_2_][PF_6_]_2_. In the case of [Cu_2_(**3c**)_2_][PF_6_]_2_, the sample did not fly in the ESMS. ^1^H-NMR spectra recorded in CD_2_Cl_2_ for [Cu_2_(**3a-c**)_2_][PF_6_]_2_, all show broad peaks in the aromatic region suggesting that more than one species is present. As previously described [18,20,21] these dinuclear double stranded arrays can exist as helicate (rac-isomer) and box (meso-isomer) and these two are commonly interchanging in the NMR timescale leading to broad NMR peaks. The meso-isomer is usually the enthalpic product (the dominant solid state species) while the helicate is favoured entropically and is the major product in solution at room temperature [18,20,21]. In particular, [Cu_2_(**3c**)_2_][PF_6_]_2_, the NMR peaks were extremely broad making peak assignments difficult. 

Liquid crystal properties of these free ligands **1a-d, 2a-d, 3a-c, and 4a-d** and the three copper Cu (I) complexes [Cu_2_(**3a-c**)_2_][PF_6_]_2_ were investigated using an OPM. Unfortunately, none of the compounds exhibited liquid crystalline phases. Possible reasons for this could lie in the ligands having too much flexibility about the central methylene unit. Secondly the two isomeric forms of the copper (I) complexes may prevent the molecules from becoming sufficiently ordered in the mesophase. 

## 3. Experimental 

### 3.1. General 

The starting materials which were commercially available were purchased from Aldrich and used as received. The solvents were purchased from Fisher Scientific (CH_2_Cl_2_, EtOAc, Et_2_O and MeOH) or Aldrich (anhydrous EtOH). Solvents were either used as received or dried; DCM was distilled from CaCl_2_ under a N_2_ atmosphere. Yields refer to chromatographically pure products. Thin-layer chromatography (TLC) was carried out on aluminum sheets coated with silica gel 60 (Merck 5554 mesh). Column chromatography was performed on silica gel 60 (Merck 230-400). Microanalyses were performed by the University of London microanalytical laboratories or by the University of Birmingham microanalytical services. Electron impact (EI) mass spectra were recorded at 70eV on a VG ProSpec mass spectrometer. Liquid secondary ion mass spectra (LSIMS) were recorded on a VG ZaBSpec mass spectrometer equipped with a cesium ion source and utilizing *m*-nitrobenzyl alcohol containing a trace of sodium acetate as the liquid matrix. ^1^H-NMR spectra were recorded on a Bruker AC 300 (300 MHz) spectrometer. ^13^C-NMR spectra were recorded on a Bruker AC 300 (75.5 MHz) spectrometer. The chemical shift values are expressed as δ values and the coupling constant values (*J*) are in Hertz (Hz). The following abbreviations are used for the signal multiplicities or characteristics: s, singlet; d, doublet; dd, doublet of doublets; t, triplet; m, muliplet; q, quartet; quint; quintet; br, broad. Transition temperatures were measured using a Mettler FP82 HT hot stage and central processor in conjunction with Leitz DMFRT Polarizing microscope as well as differential scanning calorimetry (DSC7 Perkin-Elmer).

*Methyl 3,4-dihydroxybenzoate* (**8**): A solution of 3,4-dihydroxybenzoic acid (**6**, 15.00 g, 97.30 mmol) and conc. H_2_SO_4_ (1.5 mL) in MeOH (200 mL) was stirred and heated under reflux overnight. The resultant yellow solution was allowed to cool to room temperature and the solvent removed *in vacuo*, affording a brown solid (14.60 g, 82%). mp: 126-129 °C; ^1^H-NMR (acetone-d_6_): δ_H_ 8.55 (br s, 1H, OH), 7.50 (d, *J* = 2.0 Hz, 1H, Ar-H), 7.44 (dd, 1H, Ar-H, *J* = 8.3, 2.0 Hz), 6.90 (d, 1H, Ar-H, *J* = 8.3 Hz,), 3.80 (s, 3H, OMe), 3.01 (br s, 1H, OH); ^13^C-NMR (acetone-d_6_): δ_C_ 167.0, 150.7, 145.5, 123.2, 122.7, 117.0, 115.7, 51.8; MS m/z (EIMS): 168 ([M]^+^, 54%), 137 ([M-OMe]^+^, 100%), 109 ([M-CO_2_Me]^+^, 20%); IR ν/cm^-1^ (nujol): 3468, 3263, 2927, 2854, 1690.

*Methyl 3,4,5-trihydroxybenzoate* (**9**): The same procedure as described for the preparation of **8** was followed, using 3,4,5-trihydroxybenzoic acid (7, 16.60 g, 97.40 mmol), conc. H_2_SO_4_ (0.5 mL) and MeOH (200 mL). This yielded a pale brown solid (14.40 g, 80%). mp: 120-124 °C; ^1^H-NMR (acetone-d_6_): δ_H_ 7.11 (s, 2H, Ar-H), 3.78 (s, 3H, OMe); ^13^C-NMR (acetone-d_6_): δ_C_ 167.9, 146.7, 139.5, 122.5, 110.5, 49.3; MS m/z (EIMS): 184 ([M]^+^, 55%), 153 ([M-OMe]^+^, 100%), 125 ([M-CO_2_Me]^+^, 17%); IR ν/cm^-1^ (nujol): 3468, 3263, 1690.

*Methyl 3,4-dipentyloxybenzoate* (**10a**): A suspension of K_2_CO_3_ (9.90 g, 71.63 mmol) in a solution of methyl 3,4-dihydroxybenzoate (**8**, 3.00 g, 17.84 mmol) and *n*-bromopentane (6.75 g, 44.69 mmol) in MeCN (100 mL) was stirred and heated under reflux overnight. The resultant brown mixture was allowed to cool to room temperature and concentrated *in vacuo* (30 mL). Water (250 mL) was added and the aqueous layer was extracted by washing with Et_2_O (3 x 100 mL). The combined organic layers were washed with brine solution (50 mL), dried (MgSO_4_), filtered and solvent removed *in vacuo,* yielding an orange oil as the crude product. The crude product was purified via silica gel column chromatography (gradient elution: 0 to 10 % EtOAc in hexane, increase polarity in increments of 5% per 150 mL of eluent used). The solvent was removed *in vacuo* to yield a colourless oil (4.84 g, 88%). ^1^H-NMR (CDCl_3_): δ_H_ 7.62 (dd, 1H, Ar-H, *J* = 8.5, 2.2 Hz), 7.53 (d, , 1H, Ar-H, *J* *=* 2.2 Hz), 6.86 (d, 1H, Ar-H, *J* = 8.5 Hz), 4.05-4.02 (m, 4H, 2 x OCH_2_), 3.88 (s, 3H, OMe), 1.89-1.79 (m, 4H, 2 x CH_2_), 1.50-1.33 (m, 8H, 4 x CH_2_), 0.87 (t, 6H, 2 x CH_3_, *J* = 7.0Hz); ^13^C-NMR (CDCl_3_): δ_C_ 167.0, 153.1, 148.4, 123.5, 122.4, 114.1, 111.9, 69.2, 69.0, 51.9, 28.9, 28.2, 22.5, 14.1; MS m/z (EIMS): 308 ([M]^+^, 28 %), 168 ([M-(2 x C_5_H_10_)]^+^, 137 ([M-(2 x C_5_H_10_)-OMe]^+^, 24%); IR ν/cm^-1^ (nujol): 2926, 2856, 1718.

Using the same general procedure as described for **10a**, the following compounds were prepared:

*Methyl 3,4-diheptyloxybenzoate* (**10b**): From K_2_CO_3_ (9.90 g, 71.63 mmol) methyl 3,4-dihydroxy-=benzoate (**8**, 3.00 g, 17.84 mmol) and *n*-bromoheptane (8.10 g, 45.23 mmol). This yielded a pale yellow oil (6.00 g, 92%). mp: 30-32 °C; ^1^H-NMR (CDCl_3_): δ_H_ 7.63 (dd, 1H, Ar-H, *J* = 8.5, 2.0 Hz), 7.53 (d, 1H, Ar-H, *J* = 2.0 Hz), 6.86 (d, 1H, Ar-H, *J* = 8.5 Hz), 4.05- 4.03 (m, 4H, 2 x OCH_2_), 3.88 (s, 3H, OMe), 1.85-1.80 (m, 4H, 2 x CH_2_), 1.31-1.25 (m, 16H, 8 x CH_2_), 0.88 (t, 6H, 2 x CH_3_, *J* = 6.6 Hz); ^13^C-NMR (CDCl_3_): δ_C_ 167.2, 153.3, 148.6, 123.7, 122.5, 114.3, 112.0, 69.4, 69.1, 52.1, 32.0, 29.2, 26.1, 22.8, 14.2; MS m/z (EIMS): 365 ([M+H]^+^, 100%); IR ν/cm^-1^ (nujol): 2926, 2856, 1723.

*Methyl 3,4-dinonyloxybenzoate* (**10c**): From K_2_CO_3_ (9.90 g, 71.63 mmol), methyl 3,4-dihydroxy-benzoate (**8**, 3.00 g, 17.84 mmol) and *n*-bromononane (9.26 g, 44.70 mmol). This yielded a white solid (6.64 g, 88%). mp: 36-38 °C ^1^H-NMR (CDCl_3_): δ_H_ 7.62 (dd, 1H, Ar-H, *J* = 8.5, 2.0 Hz), 7.52 (d, 1H, Ar-H, *J* = 2.0 Hz), 6.85 (d, 1H, Ar-H, *J* = 8.5 Hz), 4.05-4.03 (m, 4H, 2 x OCH_2_), 3.87 (s, 3H, OMe), 1.88-1.76 (m, 4H, 2 x CH_2_), 1.46-1.25 (m, 24H, 12 x CH_2_), 0.87 (t, 6H, 2 x CH_3_, *J* = 6.6 Hz); ^13^C-NMR (CDCl_3_): δ_C_ 167.0, 153.2, 148.5, 123.5, 122.4, 114.2, 111.9, 69.3, 69.0, 51.9, 31.9, 29.6, 29.4, 29.3, 29.1, 26.0, 22.7, 14.1; MS m/z (EIMS): 420 ([M]^+^, 46%), 154 ([M-(2 x C_7_H_14_)]^+^, 100%); IR ν/cm^-1^ (nujol): 2927, 2853, 1723. 

*Methyl 3,4-didodecanyloxybenzoate* (**10d**): From K_2_CO_3_ (9.90 g, 71.63 mmol), methyl 3,4-dihydroxy-benzoate (**8**, 3.00 g, 17.84 mmol) and *n*-bromododecane (11.12 g, 44.62 mmol). This yielded a white solid (6.81 g, 76%). mp: 46-48 °C; ^1^H-NMR (CDCl_3_): δ_H_ 7.62 (dd, 1H, Ar-H, *J* = 8.5, 2.0 Hz), 7.52 (d, 1H, Ar-H, *J* = 2.0 Hz), 6.85 (d, 1H, Ar-H, *J* = 8.5 Hz), 4.05-4.03 (m, 4H, 2 x OCH_2_), 3.87 (s, 3H, OMe), 1.88-1.79 (m, 4H, 2 x CH_2_), 1.47-1.27 (m, 36H, 18 x CH_2_), 0.87 (t, 6H, 2 x CH_3_, *J* = 6.6 Hz); ^13^C-NMR (CDCl_3_): δ_C_ 167.0, 157.3, 148.5, 123.5, 122.4, 114.2, 111.9, 69.3, 69.0, 51.9, 32.0, 29.7, 29.4, 29.2, 29.1, 26.0, 22.7, 14.2; MS m/z (EIMS): 505 ([M+H]^+^, 100%); IR ν/cm^-1^ (nujol): 2926, 2855, 1718.

*Methyl 3,4,5-tripentyloxybenzoate* (**11a**): Fromg methyl 3,4,5-trihydroxybenzoate (**9**, 3.83 g, 20.83 mmol) and *n*-bromopentane (11.01 g, 72.91 mmol), K_2_CO_3_ (11.50 g, 83.3 mmol) and MeCN (100 mL). This yielded a pale yellow oil (5.07 g, 61%). ^1^H-NMR (CDCl_3_): δ_H_ 7.24 (s, 2H, Ar-H), 4.01 (t, 6H, 3 x OCH_2_, *J* = 6.6 Hz), 3.88 (s, 3H, OMe), 1.86-1.69 (m, 6H, 3 x CH_2_), 1.48-1.32 (m, 12H, 6 x CH_2_), 0.92 (t, *J* = 7.0 Hz, 3H, CH_3_), 0.91 (t, 6H, 2 x CH_3_ *J* = 7.0 Hz); ^13^C-NMR (CDCl_3_): δ_C_ 165.4, 151.3, 140. 9, 123.0, 106.5, 71.9, 67.6, 50.5, 28.4, 27.4, 26.7, 21.0, 20.9, 12.5; MS m/z (EIMS): 394 ([M]^+^, 20%), 324 ([M-C_5_H_10_]^+^, 5%), 184 ([M-(3 x C_5_H_10_)]^+^, 100%); IR ν/cm^-1^ (nujol): 2925, 2854, 1718.

*Methyl 3,4,5-triheptyloxybenzoate* (**11b**): From K_2_CO_3_ (9.90 g, 71.63 mmol) methyl 3,4,5-trihydroxy-benzoate (**9**, 3.83 g, 20.82 mmol) and *n*-bromoheptane (13.04 g, 72.85 mmol). This yielded a yellow oil (5.97 g, 60%). ^1^H-NMR (CDCl_3_): δ_H_ 7.24 (s, 2H, Ar-H), 4.00 (t, 6H, 3 x OCH_2_, *J* = 6.6 Hz), 3.88 (s, 3H, OMe), 1.85-1.68 (m, 6H, 3 x CH_2_), 1.48-1.29 (m, 24H, 12 x CH_2_), 0.88 (t, 9H, 3 x CH_3_, *J* = 7.0 Hz); ^13^C-NMR (CDCl_3_): δ_C_ 165.1, 151.0, 140.8, 122.9, 106.2, 71.6, 67.3, 50.2, 30.0, 28.5, 27.5, 27.3, 27.2, 24.2, 20.8, 12.2; MS m/z (EIMS): 478 ([M]^+^, 72 %), 380 ([M-C_7_H_14_]^+^, 25%), 184 ([M-(3 x C_7_H_14_)]^+^, 100%); IR ν/cm^-1^ (nujol): 2930, 2870, 1723.

*Methyl 3,4,5-trinonyloxybenzoate* (**11c**): From K_2_CO_3_ (9.90 g, 71.63 mmol) methyl 3,4,5-trihydroxy-benzoate (**9**, 3.83 g, 20.82 mmol) and *n*-bromononane (15.08 g, 72.85 mmol). This yielded a yellow oil (7.37 g, 63%). ^1^H-NMR (CDCl_3_): δ_H_ 7.24 (s, 2H, Ar-H), 4.00 (t, 6H, 3 x OCH_2_, *J* = 6.6 Hz), 3.88 (s, 3H, OMe), 1.85-1.68 (m, 6H, 3 x CH_2_), 1.48-1.27 (m, 36H, 18 x CH_2_), 0.88 (t, 9H, 3 x CH_3_, *J* = 7.0 Hz); ^13^C-NMR (CDCl_3_): δ_C_ 165.8, 151.3, 140.9, 122.9, 106.8, 71.9, 67.7, 54.1, 33.9, 32.3, 31.6, 31.4, 31.3, 28.1, 24.7, 16.1; MS m/z (ESMS): 585 ([M+Na]^+^, 100%); IR ν/cm^-1^ (nujol): 2930, 2871, 1723.

*Methyl 3,4,5-tridodecyloxybenzoate* (**11d**): From K_2_CO_3_ (9.90 g, 71.63 mmol) methyl 3,4,5-trihydroxy-benzoate (**9**, 3.83 g, 20.82 mmol) and *n*-bromododecane (18.14 g, 72.85 mmol). This yielded a yellow oil (9.02 g, 63%). ^1^H-NMR (CDCl_3_): δ_H_ 7.24 (s, 2H, Ar-H), 4.00 (t, 6H, 3 x OCH_2_, *J* = 6.6 Hz), 3.88 (s, 3H, OMe), 1.85-1.68 (m, 6H, 3 x CH_2_), 1.48-1.29 (m, 54H, 27 x CH_2_), 0.88 (t, 9H, 3 x CH_3_, *J* = 6.99 Hz); ^13^C-NMR (CDCl_3_): δ_C_ 166.2, 152.0, 141.2, 123.1, 107.2, 72.6, 68.3, 51.0, 31.0, 29.4, 28.2, 28.4, 28.1, 25.0, 21.8, 13.2; MS m/z (ESMS): 688 ([M+Na]^+^, 100%); IR ν/cm^-1^ (nujol): 2930, 2870, 1723. 

*3,4-Dipentyloxybenzoic acid* (**12a**): To a solution of methyl 3,4-dipentyloxybenzoate (**10a**, 4.84 g, 15.7 mmol) in MeOH (150 mL), an aqueous solution of sodium hydroxide (15 mL, 2.10 mol L^-1^) was added and the mixture was stirred and heated under reflux overnight. The resultant colourless solution was allowed to cool to room temperature and HCl (2 M) was added dropwise to acidify the solution, whereupon a white precipitate was formed, which was filtered off. The filtrate was concentrated *in vacuo* (~20 mL) and water (100 mL) was added. The aqueous layer was extracted with Et_2_OAc (3 x 100 mL). The combined organic layers were washed with brine solution (20 mL), dried (MgSO_4_) and filtered. The solvent was removed *in vacuo*, yielding a white solid (4.00 g, 87%). mp: 126-128 °C; ^1^H- NMR (CDCl_3_): δ_H_ 7.72 (dd, 1H, Ar-H, *J* = 8.5, 2.2 Hz), 7.58 (d, 1H, Ar-H, *J* = 2.2 Hz), 6.89 (d, 1H, Ar-H, *J* = 8.5 Hz), 4.07-4.04 (m, 4H, 2 x OCH_2_), 1.88-1.82 (m, 4H, 2 x CH_2_), 1.49-1.38 (m, 8H, 4 x CH_2_), 0.87 (t, 6H, 2 x CH_3_, *J* = 6.6 Hz); ^13^C-NMR (CDCl_3_): δ_C_ 171.7, 153.9, 148.5, 124.5, 121.4, 114.5, 111.8, 69.3, 69.0, 28.7, 28.2, 22.5, 14.1; MS m/z (EIMS): 294 ([M]^+^, 25%), 224 ([M-C_5_H_10_]^+^, 9%), 154 ([M-(2 x C_5_H_10_)]^+^; IR ν/cm^-1^ (nujol): 2926, 2856, 2646, 1684. 

Following the same general procedure described for **12a**, the following compounds were prepared:

*3,4-Diheptyloxybenzoic acid* (**12b**): From compound **11b** (5.94 g, 16.30 mmol) and sodium hydroxide (15 mL, 2.18 mol L^-1^). This yielded a white solid (4.70 g, 82%). mp: 120-123 °C; ^1^H-NMR (CDCl_3_): δ_H_ 7.70 (dd, 1H, Ar-H, *J* = 8.5, 2.2 Hz), 7.56 (d, 1H, Ar-H, *J =* 2.2 Hz), 6.87 (d, 1H, Ar-H, *J* = 8.5 Hz), 4.05-4.02 (m, 4H, 2 x OCH_2_), 1.83-1.81 (m, 4H, 2 x CH_2_), 1.31-1.25 (m, 16H, 8 x CH_2_), 0.88 (t, 6H, 2 x CH_3_, *J* = 6.6 Hz); ^13^C-NMR (CDCl_3_): δ_C_ 171.9, 153.7, 148.3, 124.3, 121.3, 114.3, 111.6, 69.1, 68.8, 31.0, 28.9, 25.8, 22.8, 13.9; MS m/z (EIMS): 351 ([M+H]^+^, 25%), 333 ([M-OH]^+^, 100%); IR ν/cm^-1^ (nujol): 2925, 2855, 2646, 1670s. 

*3,4-Dinonyloxybenzoic acid* (**12c**): From compound **10c** (6.42 g, 15.26 mmol) and sodium hydroxide (15 mL, 2.03 mol L^-1^). This yielded a white solid (5.85 g, 94%). mp: 112-114°C; ^1^H-NMR (CDCl_3_): δ_H_ 7.71 (dd, 1H, Ar-H, *J* = 8.5, 2.2 Hz), 7.52 (d, 1H, Ar-H, *J* = 2.2 Hz), 6.85 (d, 1H, Ar-H, *J* = 8.5 Hz), 4.07-4.03 (m, 4H, 2 x OCH_2_), 1.87–1.79 (m, 4H, 2 x CH_2_), 1.49–1.27 (m, 24H, 12 x CH_2_), 0.87 (t, 6H, 2 x CH_3_, *J* = 6.6 Hz); ^13^C-NMR (CDCl_3_): δ_C_ 172.0, 154.1, 148.7, 124.7, 121.5, 114.6, 112.0, 69.4, 69.2, 32.1, 29.7, 29.6, 26.5, 26.2, 22.9, 14.3; MS m/z (EIMS): 406 ([M]^+^, 46%), 280 ([M-C_7_H_14_]^+^, 154 ([M-(2 x C_7_H_14_)]^+^, 100%); IR ν/cm^-1^ (nujol): 2926, 2854, 2644, 1670.

*3,4-Didodecanyloxybenzoic acid* (**12d**): From compound **10d** (6.55 g, 12.98 mmol) and sodium hydroxide (15 mL, 1.87 mol L^-1^). This yielded a white solid (5.64 g, 89%). mp: 106-109 °C; ^1^H-NMR (CDCl_3_): δ_H_ 7.71 (dd, 1H, Ar-H, *J* = 8.5, 2.2 Hz), 7.57 (d, 1H, Ar-H, *J* = 2.2 Hz), 6.88 (d, 1H, Ar-H, *J* = 8.5 Hz), 4.07-4.03 (m, 4H, 2 x OCH_2_), 1.87-1.79 (m, 4H, 2 X CH_2_), 1.52-1.26 (m, 36H, 18 x CH_2_), 0.87 (t, 6H, 2 x CH_3_ *J* = 6.6 Hz); ^13^C-NMR (CDCl_3_): δ_C_ 172.1, 154.3, 148.8, 124.9, 121.6, 114.7, 112.1, 69.6, 69.4, 32.3, 29.9, 29.8, 26.5, 26,4, 26.2, 25.6, 22.9, 14.5; MS m/z (ESMS): 513 ([M+Na]^+^, 100%); IR ν/cm^-1^ (nujol): 2926, 2854, 2644, 1670. 

*3,4,5-Tripentyloxybenzoic acid* (**13a**): From compound **11a** (4.62 g, 11.73 mmol) and sodium hydroxide (15 mL, 2.18 mol L^-1^). This yielded a white solid (4.46 g, 74%). mp: 48-50 °C; ^1^H-NMR (CDCl_3_): δ_H_ 7.12 (s, 2H, Ar-H), 3.89 (t, 2H, OCH_2_, *J* = 6.4 Hz), 3.79 (t, 4H, 2 x OCH_2_, *J* =5.9 Hz), 1.61-1.69 (m, 6H, 3 x CH_2_), 1.42-1.25 (m, 12H, 6 x CH_2_), 0.89 (t, 3H, CH_3_, *J* = 7.0 Hz), 0.85 (t, 6H, 2 x CH_3_, *J* = 6.8 Hz); ^13^C-NMR (CDCl_3_): δ_C_; 173.4, 153.5, 142.7, 127.4, 108.9, 74.1, 69.7, 31.0, 30.1, 29.3, 29.2, 23.5, 14.9; MS m/z (EIMS): 380 ([M]^+^, 25%), 170 ([M-(3 x C_5_H_11_)]^+^, 100 %); IR ν/cm^-1^ (nujol): 2927, 2854, 2646, 1684.

*3,4,5-Triheptyloxybenzoic acid* (**13b**): From compound **11b** (5.50 g, 11.51 mmol) and sodium hydroxide (15 mL, 2.18 mol L^-1^). This yielded a white solid (4.02 g, 76%). mp: 47-49 °C; ^1^H-NMR (CDCl_3_): δ_H_ 7.31 (s, 2H, Ar-H), 4.03 (t, 2H, OCH_2_, *J* = 6.6 Hz), 4.01 (t, 4H, 2 x OCH_2_, *J* = 6.6 Hz), 1.86-1.70 (m, 6H, 3 x CH_2_), 1.52-1.29 (m, 24H, 12 x CH_2_), 0.88 (t, 9H, 3 x CH_3_, *J =* 6.6 Hz,); ^13^C-NMR (CDCl_3_): δ_C_ 173.8, 153.5, 142.9, 127.6, 109.0, 74.3, 69.8, 329, 32.8, 31.4, 30.4, 30.2, 27.2, 27.0, 23.6, 15.0; MS m/z (ESMS): 487 ([M+Na]^+^, 100%); IR ν/cm^-1^ (nujol): 2926, 2854, 2646, 1670.

*3,4,5-Trinonyloxybenzoic acid* (**13c**): From compound **11c** (7.10 g, 12.63 mmol) and sodium hydroxide (15 mL, 1.68 mol dm^-3^). This yielded a white solid (5.40 g, 78%). mp: 47-49 °C; ^1^H-NMR (CDCl_3_): δ_H_ 7.30 (s, 2H, Ar-H), 4.03 (t, 2H, OCH_2_, *J* = 6.6 Hz), 4.01 (t, , 4H, 2 x OCH_2_, *J =* 6.6 Hz), 1.86-1.69 (m, 6H, 3 x CH_2_), 1.47-1.27 (m, 36H, 18 x CH_2_), 0.87 (t, 9H, 3 x CH_3_, *J =* 6.6 Hz,); ^13^C-NMR (CDCl_3_): δ_C_ 170.8, 150.8, 140.1, 121.6, 106.5, 71.5, 67.1, 29.8, 28.2, 27.6, 27.5, 27.3, 27.2, 24.0, 20,6, 12.0; MS m/z (ESMS): 547 ([M-H]^-^, 100%); IR ν/cm^-1^ (nujol): 2928, 2855, 2647, 1670.

*3,4,5-Tridodecyloxybenzoic acid* (**13d**): From compound **11d** (8.80 g, 12.79 mmol) and sodium hydroxide (15 mL, 1.71 mol L^-1^). This yielded a white solid (6.71 g, 81%). mp: 47-49 °C; ^1^H-NMR (CDCl_3_): δ_H_ 7.30 (s, 2H, Ar-H), 4.03 (t, 2H, OCH_2_, *J =* 6.6 Hz), 4.01 (t, 4H, 2 x OCH_2_, *J =* 6.6 Hz), 1.86-1.69 (m, 6H, 3 x CH_2_), 1.47-1.25 (m, 54H, 27 x CH_2_), 0.87 (t, 9H, 3 x CH_3_, *J =* 6.6 Hz); ^13^C-NMR (CDCl_3_): δ_C_ 171.3, 151.1, 141.1, 122.1, 106.8, 71.8, 67.4, 30.2, 28.6, 27.9, 27.6, 27.5, 24.3, 20.9, 12.3; MS m/z (ESMS): 697 ([M+Na]^+^, 100%); IR ν/cm^-1^ (nujol): 2926, 2854, 1674s.

*5-Hydroxy-2-methylpyridine N-oxide* (**15**): A solution of 5-hydroxy-2-methylpyridine (**14**, 15.0 g, 0.14 mol) and hydrogen peroxide 27.5 % (33 mL, 0.27 mol) in CH_3_CO_2_H (300 mL) was heated at 80 °C for 2.5 hrs. A colour change of solution was observed from orange to pale yellow. The solution was further stirred at room temperature for 24 hrs and concentrated *in vacuo* (50 mL). Acetone (400 mL) was added to force precipitation. The resulting pale yellow precipitate was collected by vacuum filtration (12.7 g, 74%). mp: 188.9-189.5 °C (decomposes); ^1^H-NMR (CD_3_OD): δ_H_ 7.93 (d, 1H, Py-H, *J* = 2.2 Hz), 7.32 (d, 1H, Py-H, *J* = 8.8 Hz), 7.03 (dd, 1H, Py-H, *J* = 8.8, 2.2 Hz), 2.41 (s, 3H, CH_3_); ^13^C-NMR (CD_3_OD): δ_C_ 158.7, 144.3, 136.0, 130.6, 122.1, 19.1; MS m/z (EIMS): 125 ([M]^+^, 50%), 109 ([M-O]^+^, 100%); IR ν/cm^-1^ (nujol): 2924, 2854, 1840, 1612.

*2-Pyridylmethanol acetate* (**16**): 5-Hydroxy-2-methylpyridine N-oxide (**15**, 17.5 g, 0.14 mol) was slowly added to (CH_3_CO)_2_O (170 mL) at 110 °C. The resultant dark brown reaction mixture was stirred for 2 hrs at 140 °C. EtOH (400 mL) was added and the solution was concentrated *in vacuo* to yield brown oil. CHCl_3_ (100 mL) was added and the solution was neutralised with saturated NaHCO_3_. The organic layers were collected through washings with saturated NaHCO_3_ (2 x 20 mL), dried (MgSO_4_) and filtered. The solvent was removed *in vacuo* to yield a brown oil, which was further dried under high vacuum for 2 hrs (26.1 g, 89%). ^1^H-NMR (CDCl_3_): δ_H_ 8.40 (d, 1H, Py-H, *J* = 2.6 Hz), 7.49 (d, 1H, Py-H, *J* = 8.5 Hz), 7.39 (dd, 1H, Py-H, *J* = 8.5, 2.6 Hz), 5.21 (s, 2H, CH_2_), 2.33 (s, 3H, CH_3_), 2.15 (s, 3H, CH_3_); ^13^C-NMR (CDCl_3_): δ_C_ 170.7, 168.9, 152.9, 146.7, 143.0, 130.0, 122.5, 66.3, 22.2, 21.0; MS m/z (FABMS): 210 ([MH]^+^, 100%); IR ν/cm^-1^ (nujol): 2923, 2854, 1747, 1582.

*5-Hydroxy-2-hydroxymethylpyridine (17):* A solution of 2-pyridylmethanol acetate (**16**) in conc. HCl (30 mL) was heated under reflux for 1.5 hrs. The reaction mixture was cooled to room temperature and concentrated *in vacuo*. CHCl_3_ (200 mL) was added and the solution neutralised carefully with saturated NaHCO_3_. The aqueous layers were collected through washings with saturated NaHCO_3_ and concentrated *in vacuo*. The brown solid was triturated with MeOH (500 mL) and filtrates collected through filtration. The solvent was removed *in vacuo* yielding a brown solid (8.3 g, 98%). ^1^H-NMR (CDCl_3_): δ_H_ 7.85 (d, 1H, Py-H, *J* = 2.6 Hz), 7.18 (d, 1H, Py-H, *J* = 8.5 Hz), 7.01 (dd, 1H, Py-H, *J* = 8.5, 2.6 Hz), 4.51 (s, 2H, CH_2_); ^13^C-NMR (CDCl_3_): δ_C_ 163.5, 147.8, 141.5, 128.0, 124.8, 66.7; MS m/z (FABMS): 125 ([M+H]^+^, 35%).

*5-Hydroxypyridine 2-carbaldehyde* (**18**): A suspension of 5-hydroxy-2-hydroxymethylpyridine (**17**, 2.50 g, 19.98 mmol) and activated MnO_2_ (1.74 g, 19.98 mmol) in *iso*-PrOH (100 mL) was heated under reflux for 3 hrs. The hot mixture was filtered through Celite^®^ and washed thoroughly with hot MeOH (400 mL). The solution was allowed to cool to room temperature and concentrated *in vacuo,* yielding a brown solid. The solid was absorbed onto silica and purified via short silica gel column chromatography (gradient elution: 0 to 5% methanol in CH_2_Cl_2_, increase polarity in increments of 2 % per 150 mL of eluent used). The solvent was removed *in vacuo* to yield a brown solid (1.41 g, 57%). mp: 223-225 °C (decomposes); ^1^H-NMR (CD_3_OD): δ_H_ 9.60 (s, 1H, CHO), 7.90 (d, 1H, Py-H, *J* = 2.2 Hz), 7.74 (d, 1H, Py-H, *J* = 8.8 Hz), 6.84 (dd, 1H, Py-H, *J* = 8.8, 2.2 Hz), ^13^C-NMR (CD_3_OD): 193.0, 172.2, 146.5, 140.9, 130.2, 126.6; MS m/z (FABMS): 123 ([M]^+^, 50%), 109 ([M-CH_2_], 27%), 95 ([M-CO]^+^, 100%); IR ν/cm^-1^ (nujol): 2923, 2854, 1668, 1507.

*3,4-Bisdipentyloxybenzoic acid 6-formylpyridin-3-ester* (**19a**): To a solution of 5-hydroxypyridine-2-carbaldehyde (**18**, 0.50 g, 2.44 mmol) in dry CH_2_Cl_2_ (30 mL), cooled in an ice bath under a N_2_ atmosphere, compound **12a** (0.72 g, 2.44 mmol), 1-(3-dimethylaminopropyl)-3-ethylcarbodiimide hydrochloride (EDC) (1.16 g, 60.55 mmol) and 4-dimethylaminopyridine (catalytical amount) were added. The solution was stirred at room temperature under a N_2_ atmosphere for 24 hrs. HCl (50 mL, 1 mol dm^-3^) was added, which yielded a white solid. MeOH (10 mL) was added to dissolve the precipitate. The organic layers were collected through washings with CH_2_Cl_2_ (3 x 30 mL), dried (MgSO_4_) and filtered. The solvent was removed *in vacuo*, to yield a brown solid. The solid was absorbed onto silica and purified via silica gel column chromatography (gradient elution: 0 to 10 % EtOAc in hexane, increase polarity in increments of 5% per 150 mL of eluent used). The solvent was removed *in vacuo* to yield a white solid (0.35 g, 36%); mp: 63-66 °C; ^1^H-NMR (CDCl_3_): δ_H_ 10.09 (s, 1H, CHO), 8.71 (d, 1H, Py-H, *J* = 8.5 Hz), 8.07 (d, 1H, Py-H, *J* = 2.6 Hz), 7.83 (dd, 1H, Ar-H, *J* = 8.5, 2.2 Hz), 7.79 (dd, 1H, Py-H, *J* = 8.5, 2.6 Hz), 7.64 (d, 1H, Ar-H, *J* = 2.2 Hz), 6.94 (d, 1H, Ar-H, *J* = 8.5 Hz), 4.09 (t, 2H, OCH_2_, *J* = 6.6 Hz), 4.07 (t, 2H, OCH_2_, *J* = 6.6 Hz) 1.92-1.82 (m, 4H, 2 x CH_2_), 1.48-1.39 (m, 8H, 4 x CH_2_), 0.94 (t, 3H, CH_3_, *J* = 7.0 Hz), 0.93 (t, 3H, CH_3_, *J* = 7.0 Hz); ^13^C-NMR (CDCl_3_): δ_C_ 192.1, 164.8, 155.4, 151.89, 150.8, 149.7, 145.0, 131.0, 125.7, 123.4, 120.9, 115.5, 112.8, 70.2, 70.0, 29.6, 29.5, 29.0, 23.3, 14.8; MS m/z (ESMS): 399 ([M]^+^, 20%), 277 ([M-OpyCO]^+^, 100%); IR ν/cm^-1^ (nujol): 2924, 2854, 1728. 

Following the same general procedure described for **19a**, the following compounds were prepared: 

*3,4-Bisdiheptyloxybenzoic acid 6-formylpyridin-3-ester* (**19b**): From compound **18** (0.50 g, 2.44 mmol), compound **12b** (0.85 g, 2.44 mmol) and EDC (1.16 g, 60.55 mmol) in dry CH_2_Cl_2_ (50 mL) to yield a white solid (0.40 g, 40%); mp: 66-68 °C; ^1^H-NMR (CDCl_3_): δ_H_ 10.08 (s, 1H, CHO), 8.70 (d, 1H, Py-H, *J* = 2.6 Hz), 8.07 (d, 1H, Py-H, *J* = 8.5 Hz), 7.82 (dd, 1H, Ar-H, *J* = 8.5, 2.2 Hz), 7.79 (dd, 1H, Py-H, *J* = 8.5, 2.6 Hz), 7.64 (d, 1H, Ar-H, *J* = 2.2 Hz), 6.95 (d, 1H, Ar-H, *J* = 8.5 Hz), 4.09 (t, 2H, OCH_2_, *J* = 6.6 Hz), 4.06 (t, 2H, OCH_2_, *J* = 6.6 Hz), 1.89-1.83 (m, 4H, 2 x CH_2_), 1.49-1.30 (m, 16H, 8 x CH_2_), 0.89-0.84 (m, 6H, 2 x CH_3_); ^13^C-NMR (CDCl_3_): δ_C_ 192.2, 164.6, 152.7, 149.1, 148.1, 147.0, 142.3, 128.3, 123.0, 120.7, 118.2, 112.8, 110.1, 67.6, 67.3, 29.9, 27.3, 27.1, 24.1, 20.7, 12.2; MS m/z (FABMS): 478 ([M+Na]^+^, 25%); IR ν/cm^-1^ (nujol): 2925, 2854, 1728.

*3,4-Bisdinonyloxybenzoic acid 6-formylpyridin-3-ester* (**19c**): From compound **18** (0.50 g, 2.44 mmol), compound **12c** (0.99 g, 2.44 mmol) and EDC (1.16 g, 60.55 mmol) in dry CH_2_Cl_2_ (50 mL). This yielded a white solid (0.45 g, 36%); mp: 68-70 °C; ^1^H-NMR (CDCl_3_): δ_H_ 10.08 (s, 1H, CHO), 8.71 (d, 1H, Py-H, *J* = 2.2 Hz), 8.07 (d, 1H, Py-H, *J* = 8.5 Hz), 7.83 (dd, 1H, Ar-H, *J* = 8.5, 2.2 Hz), 7.79 (dd, 1H, Py-H, *J* = 8.5, 2.2 Hz), 7.64 (d, 1H, Ar-H, *J* = 2.2 Hz), 6.94 (d, 1H, Ar-H, *J* = 8.5 Hz), 4.09 (t, 2H, OCH_2_, *J* = 6.6 Hz), 4.06 (t, 2H, OCH_2_, *J* = 6.6 Hz), 1.89-1.82 (m, 4H, 2 x CH_2_), 1.49-1.28 (m, 24H, 12 x CH_2_), 0.89-0.85 (m, 6H, 2 x CH_3_); ^13^C-NMR (CDCl_3_): δ_C_ 192.1, 162.5, 153.7, 149.5, 148.5, 147.4, 142.7, 128.7, 123.4, 121.1, 118.6, 113.2, 110.5, 68.0, 67.7, 30.4, 28.1, 27.9, 27.8, 27.5, 24.5, 21.2, 12.6; MS m/z (ESMS): 534 ([M+Na]^+^, 85%); IR ν/cm^-1^ (nujol): 2930, 2855, 1730.

*3,4-Bisdidodecyloxybenzoic acid 6-formylpyridin-3-ester* (**19d**): From compound **18** (0.50 mg, 2.44 mmol), compound **12d** (1.20 g, 2.44 mmol) and EDC (1.16 g, 60.55 mmol) in dry CH_2_Cl_2_ (50 mL). This yielded a white solid (0.46 g, 32%); mp: 70-71 °C: ^1^H-NMR (CDCl_3_): δ_H_ 10.09 (s, 1H, CHO), 8.71 (d, 1H, Py-H, *J* = 2.6 Hz), 8.71 (d, 1H, Py-H, *J* = 8.5 Hz), 7.83 (dd, 1H, Ar-H, *J* = 8.5, 2.2 Hz), 7.79 (dd, 1H, Py-H, *J* = 8.5, 2.6 Hz), 7.64 (d, 1H, Ar-H, *J* = 2.2 Hz), 6.95 (d, 1H, Ar-H, *J* = 8.5 Hz), 4.09 (t, 2H, OCH_2_, *J* = 6.6 Hz), 4.07 (t, 2H, OCH_2_, *J* = 6.6 Hz), 1.96-1.82 (m, 4H, 2 x CH_2_), 1.49-1.26 (m, 36H, 18 x CH_2_), 0.87 (t, 6H, 2 x CH_3_, *J* = 6.6 Hz); ^13^C-NMR (CDCl_3_): δ_C_ 192.1, 164.0, 154.5, 150.9, 149.9, 148.8, 144.2, 130.2, 124.8, 122.5, 120.0, 114.5, 111.9, 69.4, 69.1, 31.9, 29.6, 29.4, 29.1, 26.0, 22.7, 14.1; MS m/z (ESMS): 618 ([M+Na]^+^, 100%); IR ν/cm^-1^ (nujol): 2929, 2856, 1731.

*3,4,5-Trispentyloxybenzoic acid 6-formylpyridin-3-ester* (**20a**): From compound **18** (0.50 g, 4.07 mmol), compound **13a** (1.55 g, 4.07 mmol) and EDC (1.16 g, 6.06 mmol) in dry CH_2_Cl_2_ (50 mL). This yielded a brown oil (0.77 g, 40%); ^1^H-NMR (CDCl_3_): δ_H_ 10.09 (s, 1H, CHO), 8.70 (d, 1H, Py-H, *J* = 2.6 Hz), 8.08 (d, 1H, Py-H, *J* = 8.5 Hz), 7.77 (dd, 1H, Py-H, *J* = 8.5, 2.6 Hz), 7.39 (s, 2H, Ar-H), 4.07 (t, 2H, OCH_2_, *J* = 6.6 Hz), 4.04 (t, 4H, 2 x OCH_2_, *J* = 6.6 Hz) 1.89-1.72 (m, 6H, 3 x CH_2_), 1.50-1.26 (m, 12H, 6 x CH_2_), 0.93 (t, 9H, 3 x CH_3_, *J* = 7.0 Hz); ^13^C-NMR (CDCl_3_): δ_C_ 192.2, 164.2, 153.3, 151.1, 150.3, 144.4, 144.0, 130.4, 122.8, 122.6, 109.0, 73.8, 69.6, 30.1, 29.1, 28.4, 22.6, 22.5, 14.1; MS m/z (EIMS): 485 ([M]^+^, 15%), 363 ([M-OPyCHO]^+^, 100%), 293 ([M-OPyCHO-C_5_H_10_]^+^, 75%), 153 ([M-OPyCHO-(3xC_5_H_10_)]^+^, 30%); IR ν/cm^-1^ (nujol): 2927, 2854, 1728.

*3,4,5-Trisheptyloxybenzoic acid 6-formylpyridin-3-ester* (**20b**): From compound **18** (0.50 g, 4.07 mmol), compound **13b** (1.89 g, 4.07 mmol) and EDC (1.16 g, 6.06 mmol) in dry CH_2_Cl_2_ (50 mL). This yielded a brown oil (0.88 g, 39%); ^1^H-NMR (CDCl_3_): δ_H_ 10.09 (s, 1H, CHO), 8.70 (d, 1H, Py-H, *J* = 2.6 Hz), 8.07 (d, 1H, Py-H, *J* = 8.5 Hz), 7.77 (dd 1H, Py-H, *J* = 8.5, 2.6 Hz), 7.39 (s, 2H, Ar-H), 4.06 (t, 2H, OCH_2_, *J* = 6.6 Hz), 4.06 (t, 4H, 2 x OCH_2_, *J* = 6.6 Hz), 1.88-1.71 (m, 6H, 3 x CH_2_), 1.50-1.30 (m, 24H, 12 x CH_2_), 0.88 (t, 9H, 3 x CH_3_, *J* = 6.4 Hz); ^13^C-NMR (CDCl_3_): δ_C_ 192.4, 164.4, 153.5, 151.3, 150.5, 144.5, 144.2, 130.6, 122.9, 122.8, 109.2, 74.0, 69.7, 32.3, 32.2, 29.6, 29.4, 26.4, 23.0, 14.4; MS m/z (ESMS): 592 ([M+Na]^+^, 100%); IR ν/cm^-1^ (nujol): 2926, 2854, 1728.

*3,4,5-Bistrinonylloxybenzoic acid 6-formylpyridin-3-ester* (**20c**): From compound **18** (0.50 g, 4.07 mmol), compound **13c** (2.23 g, 4.07 mmol) and EDC (1.16 g, 60.55 mmol) in dry CH_2_Cl_2_ (50 mL). This yielded a white solid (0.89 g, 34%); mp: 34-37 °C; ^1^H-NMR (CDCl_3_): δ_H_ 10.08 (s, 1H, CHO), 8.69 (d, 1H, Py-H, *J* = 2.6 Hz), 8.07 (d, 1H, Py-H, *J* = 8.5 Hz), 7.77 (dd, 1H, Py-H, *J* = 8.5, 2.6 Hz), 7.39 (s, 2H, Ar-H), 4.07 (t, 2H, OCH_2_, *J* = 6.4 Hz), 4.04 (t, 4H, 2 x OCH_2_, *J* = 6.6 Hz), 1.87-1.71 (m, 6H, 3 x CH_3_), 1.50-1.26 (m, 36H, 18 x CH_2_), 0.87 (t, 3H, CH_3_, *J* = 6.6 Hz), 0.86 (t, 6H, 2 x CH_3_, *J* = 7.0 Hz); ^13^C-NMR (CDCl_3_): δ_C_ 194.7, 166.7, 155.8, 153.6, 152.8, 146.8, 146.5, 132.9, 125.3, 125.1, 111.5, 76.3, 72.0, 34.5, 33.0, 32.3, 32.2, 32.0, 28.7, 25.3, 16.7; MS m/z (ESMS): 676 ([M+Na]^+^, 100%); IR ν/cm^-1^ (nujol): 2927, 2855, 1730.

*3,4,5-Trisdodecyloxybenzoic acid 6-formylpyridin-3-ester* (**20d**): From compound **18** (0.50 g, 4.07 mmol), compound **13d** (2.74 g, 2.44 mmol) and EDC (1.16 g, 60.55 mmol) in dry CH_2_Cl_2_ (50 mL). This yielded a white solid (0.89 g, 35%); mp: 41-43 °C: ^1^H-NMR (CDCl_3_): δ_H_ 10.08 (s, 1H, CHO), 8.69 (d, 1H, Py-H, *J* = 2.2 Hz), 8.07 (d, 1H, Py-H, *J* = 8.5 Hz), 7.77 (dd, 1H, Py-H, *J* = 8.5, 2.2 Hz), 7.39 (s, 2H, Ar-H), 4.06 (t, 2H, OCH_2_, *J* = 6.6 Hz), 4.04 (t, 4H, 2 x OCH_2_, *J* = 6.6 Hz), 1.85-1.71 (m, 6H, 3 x CH_2_), 1.49-1.25 (m, 54H, 27 x CH_2_), 0.86 (t, 9H, 3 x CH_3_, *J* = 6.6 Hz); ^13^C-NMR (CDCl_3_): δ_C_ 191.3, 163.3, 152.4, 150.2, 149.4, 143.4, 143.1, 129.4, 121.8, 121.6, 108.1, 72.9, 68.6, 31.2, 29.6, 28.8, 28.6, 25.3, 21.9, 13.3; MS m/z (ESMS): 802 ([M+Na]^+^, 100%), 780 ([M+H]^+^, 20%); IR ν/cm^-1^ (nujol): 2927, 2856, 1731.

*6-{N-[4-({4-[(E)-[(5-{[3, 4-bis (pentyloxy) phenyl]carbonyloxy}pyridin-2-yl)methylidene]amino] phenyl}methyl)phenyl]carboximidoyl}pyridin-3-yl 3,4-bis(pentyloxy)benzoate* (**1a**): To a solution of compound **19a** (202 mg, 0.51 mmol) in anhydrous EtOH (4 mL) under N_2_ atmosphere, 4,4’-methylene dianiline (50 mg, 0.25 mmol) was added. The reaction mixture was stirred and heated at 50 °C for 24 hrs under a N_2_ atmosphere. The solid was collected through suction filtration and recrystallised in anhydrous PhMe. The yellow solid was collected through suction filtration (140 mg, 58%); mp: 158-159 °C; Calculated for C_59_H_68_N_4_O_8_: 73.72% C, 7.13% H, 5.83% N; found: 73.68% C, 7.10% H, 5.90% N; ^1^H-NMR (CDCl_3_): δ_H_ 8.63 (s, 2H, HC=N), 8.62 (d, 2H, Py-H, *J* = 2.2 Hz), 8.28 (d, 2H, Py-H, *J* = 8.5 Hz), 7.83 (dd, 2H, Ar-H, *J* = 8.5, 1.8 Hz), 7.71 (dd, 2H, Py-H, *J* = 8.5, 2.2 Hz), 7.65 (d, 2H, Ar-H, *J* = 1.8 Hz), 7.26 (s, 8H, Ar-H), 6.94 (d, 2H, Ar-H, *J* = 8.5 Hz), 4.09 (t, 4H, 2 x OCH_2_, *J* = 6.6 Hz), 4.07 (t, 4H, 2 x OCH_2_, *J* = 6.6 Hz), 4.05 (s, 2H, Ph_2_CH_2_), 1.90-1.81 (m, 8H, 4 x CH_2_), 1.55-1.34 (m, 16H, 8 x CH_2_), 0.94 (t, 6H, 2 x CH_3_, *J* = 7.0 Hz), 0.93 (t, 6H, 2 x CH_3_, *J* = 7.0 Hz); ^13^C-NMR (CDCl_3_): δ_C_, 166.8, 161.5, 156.8, 154.4, 151.1, 151.4, 145.9, 142.3, 132.5, 132.3, 132.1, 127.2, 124.9, 123.9, 123.0, 117.1, 114.5, 71.9, 71.6, 43.5, 31.3, 31.2, 30.7, 24.5, 16.5; MS m/z (ESMS): 962 ([M+H]^+^, 100%); IR ν/cm^-1^ (nujol): 2927, 2854, 1729, 1598. 

Following the same general procedure described for the preparation of **1a**, the following compounds were prepared:

*6-{N-[4-({4-[(E)-[(5-{[3,4-bis(heptyloxy)phenyl]carbonyloxy}pyridin-2-yl)methylidene]amino]-phenyl}methyl)phenyl]carboximidoyl}pyridin-3-yl 3,4-bis(heptyloxy)benzoate* (**1b**): From compound **19b** (230 mg, 0.51 mmol), 4,4’-methylenedianiline (50 mg, 0.25 mmol) and anhydrous EtOH (4 mL). This yielded a pale yellow solid (186 mg, 69%); mp: 136-137 °C; Calculated for C_67_H_84_N_4_O_8_: 74.97% C, 7.89% H, 5.22% N; found: 74.88% C, 7.70% H, 5.18% N; ^1^H-NMR (CDCl_3_): δ_H_ 8.63 (s, 2H, HC=N), 8.62 (d, 2H, Py-H, *J* = 2.6 Hz), 8.28 (d, 2H, Py-H, *J* = 8.5 Hz), 7.83 (dd, 2H, Ar-H, *J* = 8.5, 2.1 Hz), 7.71 (dd, 2H, Py-H, *J* = 8.5, 2.6 Hz), 7.65 (d, 2H, Ar-H, *J* = 2.1 Hz), 7.26 (s, 8H, Ar-H), 6.94 (d, 2H, Ar-H, *J* = 8.5 Hz), 4.09 (t, 4H, 2 x OCH_2_, *J* = 6.6 Hz), 4.07 (t, 4H, 2 x OCH_2_, *J* = 6.6 Hz), 4.04 (s, 2H, Ph_2_CH_2_), 1.91-1.80 (m, 8H, 4 x CH_2_), 1.55-1.34 (m, 32H, 16 x CH_2_), 0.89 (t, 6H, 2 x CH_3_, *J* = 6.99 Hz), 0.88 (t, 6H, 2 x CH_3_, *J* = 6.99 Hz); ^13^C-NMR (CDCl_3_): δ_C_ 165.5, 160.1, 155.5, 153.1, 150.1, 150.0, 144.5, 140.9, 131.2, 130.9, 130.7, 125.8, 123.5, 122.5, 121.6, 115.8, 113.1, 70.5, 70.3, 42.2, 32.9, 30.3, 30.2, 27.1, 23.7, 15.2; MS m/z (ESMS): 1096 ([M+Na]^+^, 100%); IR ν/cm^-1^ (nujol): 2926, 2855, 1729, 1598. 

*6-{N-[4-({4-[(E)-[(5-{[3,4-bis(nonyloxy)phenyl]carbonyloxy}pyridin-2-yl)methylidene]amino]- phenyl}methyl)phenyl]carboximidoyl}pyridin-3-yl 3,4-bis(nonyloxy)benzoate* (**1c**): From compound **19c** (258 mg, 0.51 mmol), 4,4’-methylenedianiline (50 mg, 0.25 mmol) and anhydrous EtOH (5 mL). This yielded a pale yellow solid (155 mg, 52%); mp: 129 °C; Calculated for C_75_H_100_N_4_O_8_: 75.98% C, 8.50% H, 4.73% N; found: 75.80% C, 8.40% H, 4.63% N; ^1^H-NMR (CDCl_3_): δ_H_ 8.63 (s, 2H, HC=N), 8.62 (d, 2H, Py-H, *J* = 2.6 Hz), 8.29 (d, 2H, Py-H, *J* = 8.8 Hz), 7.83 (dd, 2H, Ar-H, *J* = 8.5, 1.8 Hz), 7.71 (dd, 2H, Py-H, *J* = 8.8, 2.6 Hz), 7.65 (d, 2H, Ar-H, *J* = 1.8 Hz), 7.26 (s, 8H, Ar-H), 6.94 (d, 2H, Ar-H, *J* = 8.5 Hz), 4.09 (t, 4H, 2 x OCH_2_, *J* = 6.6 Hz), 4.07 (t, 4H, 2 x OCH_2_, *J* = 6.6 Hz), 4.05 (s, 2H, CH_2_), 1.89-1.80 (m, 8H, 4 x CH_2_), 1.49-1.29 (m, 48H, 24 x CH_2_), 0.88 (t, 6H, 2 x CH_3_, *J* = 7.0 Hz), 0.87 (t, 6H, 2 x CH_3_, *J* = 7.0 Hz); ^13^C-NMR (CDCl_3_): δ_C_ 166.0, 160.7, 156.0, 155.2, 153.6, 150.6, 150.5, 145.0, 141.4, 131.7, 131.4, 126.4, 124.0, 123.1, 122.1, 116.3, 113.6, 71.1, 70.8, 42.8, 33.5, 31.2, 31.0, 30.9, 30.8, 30.7, 27.6, 24.3, 15.7; MS m/z (ESMS): 1207 ([M+Na]^+^); IR ν/cm^-1^ (nujol): 2926, 2854, 1727, 1598. 

*6-{N-[4-({4-[(E)-[(5-{[3,4-bis(dodecyloxy)phenyl]carbonyloxy}pyridin-2-yl)methylidene]amino]- phenyl}methyl)phenyl]carboximidoyl}pyridin-3-yl 3,4-bis(dodecyloxy)benzoate* (**1d**)**:** From compound **19d** (301 mg, 0.51 mmol), 4,4’-methylenedianiline (50 mg, 0.25 mmol) and anhydrous EtOH (6 mL). This yielded a pale yellow solid (190 mg, 56%); mp: 126 °C; Calculated for C_87_H_124_N_4_O_8_: 77.18% C, 9.23% H, 4.14% N; found: 77.23% C, 9.31% H, 4.17% N; ^1^H-NMR (CDCl_3_): δ_H_ 8.63 (s, 2H, HC=N), 8.62 (d, 2H, Py-H, *J* = 2.6 Hz,), 8.29 (d, 2H, Py-H, *J* = 8.8 Hz), 7.83 (dd, 2H, Ar-H, *J* = 8.5, 1.8 Hz), 7.71 (dd, 2H, Py-H, *J* = 8.8, 2.6 Hz), 7.65 (d, 2H, Ar-H, *J* = 1.8 Hz), 7.26 (s, 8H, Ar-H), 6.94 (d, 2H, Ar-H, *J* = 8.5 Hz), 4.09 (t, 4H, 2 x OCH_2_, *J* = 6.6 Hz), 4.07 (t, 4H, 2 x OCH_2_, *J* = 6.6 Hz), 4.05 (s, 2H, Ph_2_CH_2_), 1.89-1.80 (m, 8H, 4 x CH_2_), 1.49-1.29 (m, 72H, 36 x CH_2_), 0.88 (t, 6H, 2 x CH_3_, *J* = 7.0 Hz), 0.87 (t, 6H, 2 x CH_3_, *J* = 7.0 Hz); ^13^C-NMR (CDCl_3_): δ_C_ 165.5, 160.2, 155.6, 154.7, 153.1, 150.2, 150.1, 144.2, 141.0, 131.3, 131.1, 126.4, 123.6, 122.7, 121.7, 115.7, 110.0, 71.1, 70.8, 42.8, 33.5, 31.2, 31.0, 30.9, 30.8, 30.7, 27.6, 24.3, 15.7; MS m/z (ESMS): 1354 ([M]^+^, 100%); IR ν/cm^-1^ (nujol): 2927, 2854, 1727, 1597.

*6-{N-[4-({4-[(E)-[(5-{[3,4,5-tris(pentyloxy)phenyl]carbonyloxy}pyridin-2-yl)methylidene]amino]- phenyl}methyl)phenyl]carboximidoyl}pyridin-3-yl 3,4,5-tris(pentyloxy)benzoate* (**2a**): From compound **20a** (245 mg, 0.51 mmol), 4,4’-methylenedianiline (50 mg, 0.25 mmol) and anhydrous EtOH (4 mL). This yielded a yellow solid (151 mg, 53%). mp: 67 °C; Calculated for C_69_H_88_N_4_O_10_: 73.12% C, 7.83% H, 4.94% N; found: 73.16% C, 7.87% H, 4.89% N; ^1^H-NMR (CDCl_3_): δ_H_ 8.63 (s, 2H, HC=N), 8.61 (d, 2H, Py-H, J = 2.6 Hz), 8.29 (d, 2H, Py-H, J = 8.8 Hz), 7.71 (dd, 2H, Py-H, J = 8.8, 2.6 Hz), 7.41 (s, 4H, Ar-H), 7.26 (s, 8H, Ar-H), 4.07 (t, 4H, 2 x OCH_2_, J = 6.6 Hz), 4.05 (t, 8H, 4 x OCH_2_, J = 6.6 Hz), 4.05 (s, 2H, Ph_2_CH_2_), 1.89-1.72 (m, 12H, 6 x CH_2_), 1.53-1.31 (m, 24H, 12 x CH_2_), 0.93 (t, 18H, 6 x CH_3_, J = 7.0 Hz); ^13^C-NMR (CDCl_3_): δ_C_ 164.7, 159.3, 153.4, 152.4, 149.3, 149.1, 143.9, 143.7, 140.2, 130.4, 130.2, 123.2, 122.7, 121.8, 109.1, 74.0, 69.7, 41.5, 31.3, 29.3, 28.6, 22.9, 22.8, 14.4; MS m/z (ESMS): 1133 ([M]^+^); IR ν/cm^-1^ (nujol): 2930, 2857, 1743, 1587. 

*6-{N-[4-({4-[(E)-[(5-{[3,4,5-tris(heptyloxy)phenyl]carbonyloxy}pyridin-2-yl)methylidene]amino]- phenyl}methyl)phenyl]carboximidoyl}pyridin-3-yl 3,4,5-tris(heptyloxy)benzoate* (**2b**): From compound **20b** (287 mg, 0.51 mmol), 4,4’-methylenedianiline (50 mg, 0.25 mmol) and anhydrous EtOH (4 mL). This yielded a yellow solid (197 mg, 60%); mp: 54-56 °C; Calculated for C_81_H_112_N_4_O_10_: 74.73% C, 8.67% H, 4.30% N; found: 74.78% C, 8.70% H, 4.32% N; ^1^H-NMR (CDCl_3_): δ_H_ 8.63 (s, 2H, HC=N), 8.61 (d, 2H, Py-H, *J* = 2.6 Hz), 8.29 (d, 2H, Py-H, *J* = 8.5 Hz), 7.70 (dd, 2H, Py-H, *J* = 8.5, 2.6 Hz), 7.40 (s, 4H, Ar-H), 7.26 (s, 8H, Ar-H), 4.07 (t, 4H, 2 x OCH_2_, *J* = 6.4 Hz), 4.05 (t, 8H, 4 x OCH_2_, *J* = 6.3 Hz), 4.05 (s, 2H, Ph_2_CH_2_), 1.88-1.71 (m, 12H, 6 x CH_2_), 1.50-1.27 (m, 48H, 12 x CH_2_), 0.88 (t, 6H, 2 x CH_3_, *J* = 6.6 Hz), 0.87 (t, 12H, 4 x CH_3_, *J* = 7.0 Hz); ^13^C-NMR (CDCl_3_): δ_C_ 163.1, 157.7, 151.9, 150.1_,_ 147.7, 147.6, 142.4, 142.2, 138.6, 128.8, 128.6, 121.6, 121.2, 120.2, 107.6, 72.4, 68.2, 39.9, 30.7, 30.6, 29.1, 28.1, 28.0, 27.8, 24.8, 21.4, 12.9; MS m/z (ESMS): 1302 ([M+H]^+^); IR ν/cm^-1^ (nujol): 2930, 2857, 1744s, 1590.

*6-{N-[4-({4-[(E)-[(5-{[3,4,5-tris(nonyloxy)phenyl]carbonyloxy}pyridin-2-yl)methylidene]amino] phenyl}methyl)phenyl]carboximidoyl}pyridin-3-yl 3,4,5-tris(nonyloxy)benzoate* (**2c**): From compound **20c** (330 mg, 0.51 mmol), 4,4’-methylenedianiline (50 mg, 0.25 mmol) and anhydrous EtOH (5 mL). This yielded a yellow solid (241 mg, 65%); mp: 60-62 °C; Calculated for C_93_H_136_N_4_O_10_: 75.98% C, 9.32% H, 3.81% N; found: 75.90% C, 9.27% H, 3.85% N; ^1^H-NMR (CDCl_3_): δ_H_ 8.63 (s, 2H, HC=N), 8.61 (d, 2H, Py-H, *J* = 2.6 Hz), 8.29 (d, 2H, Py-H, *J* = 8.5 Hz), 7.70 (dd, 2H, Py-H, *J* = 8.5, 2.6 Hz), 7.40 (s, 4H, Ar-H), 7.26 (s, 8H, Ar-H), 4.07 (t, 4H, 2 x OCH_2_, *J* = 6.6 Hz), 4.05 (t, 8H, 4 x OCH_2_, *J* = 6.6 Hz), 4.05 (s, 2H, CH_2_), 1.88-1.71 (m, 12H, 6 x CH_2_), 1.50-1.21 (m, 72H, 36 x CH_2_), 0.88 (t, 6H, 2 x CH_3_, *J* = 7.0 Hz), 0.87 (t, 12H, 4 x CH_3_, *J* = 7.0 Hz); ^13^C-NMR (CDCl_3_): δ_C_ 164.4, 159.0, 153.1, 152.1, 149.0, 148.8, 143.6, 143.4, 139.9, 130.1, 129.8, 122.8, 122.4, 121.4, 108.8, 73.7, 69.4, 41.4, 31.9, 30.4, 29.7, 29.6, 29.4, 29.3, 26.1, 22.7, 14.1; MS m/z (ESMS): 1471 ([M+H]^+^); IR ν/cm^-1^ (nujol): 2930, 2857, 1744, 1589. 

*6-{N-[4-({4-[(E)-[(5-{[3,4,5-tris(dodecyloxy)phenyl]carbonyloxy}pyridin-2-yl)methylidene]amino] phenyl}methyl)phenyl]carboximidoyl}pyridin-3-yl 3,4,5-tris(dodecyloxy)benzoate* (**2d**): From compound **20d** (398 mg, 0.51 mmol), 4,4’-methylenedianiline (50 mg, 0.25 mmol) and anhydrous EtOH (6 mL). This yielded a yellow solid (291 mg, 67%); mp 51 °C; Calculated for C_111_H_172_N_4_O_10_: 77.40% C, 10.06% H, 3.25% N; found: 77.33% C, 10.01% H, 3.27% N; ^1^H-NMR (CDCl_3_): δ_H_ 8.63 (s, 2H, HC=N), 8.61 (d, 2H, Py-H, *J* = 2.6 Hz), 8.29 (d, 2H, Py-H, *J* = 8.8 Hz), 7.70 (dd, 2H, Py-H, *J* = 8.8, 2.6 Hz), 7.40 (s, 4H, Ar-H), 7.27 (s, 8H, Ar-H), 4.06 (t, 4H, 2 x OCH_2_, *J* = 6.4 Hz), 4.04 (t, 8H, 4 x OCH_2_, *J* = 6.3 Hz), 4.04 (s, 2H, Ph_2_CH_2_), 1.89-1.71 (m, 12H, 6 x CH_2_), 1.48-1.25 (m, 108H, 54 x CH_2_), 0.87 (t, 18H, 9 x CH_2_, *J* = 6.6 Hz); ^13^C-NMR (CDCl_3_): δ_C_ 162.5, 157.1, 151.2, 150.2, 147.1, 146.9, 141.7, 141.5, 138.0, 128.2, 128.0, 120.9, 120.5, 119.6, 106.9, 71.8, 67.5, 30.1, 28.5, 27.8, 27.5, 24.2, 20.8, 12.3; MS m/z (ESMS): 1744 ([M+Na]^+^); IR ν/cm^-1^ (nujol): 2930, 2857, 1729, 1598.

*6-{N-[3-({3-[(E)-[(5-{[3,4-bis(pentyloxy)phenyl]carbonyloxy}pyridin-2-yl)methylidene]amino]- phenyl}methyl)phenyl]carboximidoyl}pyridin-3-yl 3,4-bis(pentyloxy)benzoate* (**3a**): From compound **19a** (60 mg, 0.16 mmol), 3,3’-methylenedianiline (10 mg, 0.08 mmol) and anhydrous EtOH (2 mL). This yielded a brown oil (60 mg, 85%). ^1^H-NMR (CDCl_3_): δ_H_ 8.61 (s, 2H, HC=N), 8.60 (d, 2H, Py-H, *J* = 2.3 Hz), 8.27 (d, 2H, Py-H, *J* = 8.7 Hz), 7.82 (dd, 2H, Ar-H, *J* = 8.5, 2.1 Hz), 7.71 (dd, 2H, Py-H, *J* = 8.7, 2.3 Hz), 7.65 (d, 2H, Ar-H, *J* = 2.1 Hz), 7.35 (t, 2H, Ar-H, *J* = 8.5 Hz), 7.15 (m, 6H, Ar-H), 6.94 (d, 2H, Ar-H, *J* = 8.5 Hz), 4.07 (m, 10H, Ph_2_CH_2_, OCH_2_), 1.88-1.68 (m, 8H, 4 x CH_2_), 1.52-1.31 (m, 16H, 8 x CH_2_), 0.93 (t, 12H, 4 x CH_3_, *J* = 7.0 Hz); ^13^C-NMR (CDCl_3_): δ_C_ 160.0, 152.2, 151.5, 149.3, 143.8, 142.4, 130.6, 130.5, 128.8, 127.9, 125.1, 122.8, 122.4, 120.9, 119.2, 115.0, 112.3, 42.2, 29.2, 29.1, 28.6, 22.9, 14.4; MS m/z (+ve FAB): 961 ([M]^+^, 80%). 

*6-{N-[3-({3-[(E)-[(5-{[3,4-bis(heptyloxy)phenyl]carbonyloxy}pyridin-2-yl)methylidene]amino]- phenyl}methyl)phenyl]carboximidoyl}pyridin-3-yl 3,4-bis(heptyloxy)benzoate* (**3b**): From compound **19b** (50 mg, 0.11 mmol), 3,3’-methylenedianiline (10 mg, 0.06 mmol) and anhydrous EtOH (2 mL). This yielded a brown oil (40 mg, 76%). ^1^H-NMR (CDCl_3_): δ_H_ 8.61 (s, 2H, HC=N), 8.61 (d, 2H, Py-H, *J* = 2.5 Hz), 8.27 (d, 2H, Py-H, *J* = 8.6 Hz), 7.83 (dd, *J* = 2H, Ar-H, 8.3, 2.0 Hz), 7.71 (dd, *J* = 2H, Py-H, 8.6, 2.3 Hz), 7.65 (d, 2H, Ar-H, *J* = 2.0 Hz), 7.35 (t, 2H, Ar-H, 7.5 Hz), 7.16-7.13 (m, 6H, Ar-H), 6.94 (d, 2H, Ar-H, *J* = 8.3 Hz), 4.09-4.05 (m, 10H, Ph_2_CH_2_, 4 x OCH_2_), 1.88-1.71 (m, 8H, 4 x CH_2_), 1.52-1.30 (m, 32H, 16 x CH_2_), 0.93 (m, 12H, 4 x CH_3_); ^13^C-NMR (CDCl_3_): δ_C_ 160.0, 143.8, 130.5, 129.8, 127.8, 125.1, 122.8, 122.4, 119.1, 115.0, 112.4, 32.2, 29.6, 29.4, 26.4, 26.3, 23.0, 14.5; MS m/z (+ve FAB): 1073 ([M]^+^); IR ν/cm^-1^ (nujol): 2911, 2851, 1729, 1598.

*6-{N-[3-({3-[(E)-[(5-{[3,4-bis(dodecyloxy)phenyl]carbonyloxy}pyridin-2-yl)methylidene]amino]- phenyl}methyl)phenyl]carboximidoyl}pyridin-3-yl 3,4-bis(dodecyloxy)benzoate* (**3c**): From compound **19d** (70 mg, 0.12 mmol), 3,3’-methylenedianiline (10 mg, 0.06 mmol) and anhydrous EtOH (2 mL). This yielded a white solid (70 mg, 83%); Calculated for C_87_H_124_N_4_O_8_·H_2_O: 76.2% C, 9.1% H, 4.1% N; found: 75.9% C, 9.0% H, 3.9% N; ^1^H-NMR (CDCl_3_): δ_H_ 8.60 (s, 2H, CHN), 8.61 (d, 2H, Py-H, *J* = 2.0 Hz), 8.27 (d, 2H, Py-H, *J* = 8.6 Hz), 7.83 (dd, *J* = 2H, Ar-H, 8.6, 2.0 Hz), 7.71 (d, 2H, Py-H, *J* = 8.3, 2.0 Hz), 7.65 (d, 2H, Ar-H, *J* = 2.0 Hz), 7.65 (s, 2H, Ar-H), 7.34 (t, 2H, Ar-H, *J* = 7.6 Hz), 7.16-7.13 (m, 6H, Ar-H), 6.94 (d, 2H, Ar-H, *J* = 8.6 Hz), 4.09 (m, 10H, Ph_2_CH_2_, 4 x OCH_2_), 1.88-1.71 (m, 8H, 4 x CH_2_), 1.48-1.27 (m, 72H, 36 x CH_2_), 0.93 (t, 12H, 4 x CH_3_, *J* = 7.0 Hz); MS m/z (+ve FAB): 1354 ([M]^+^); IR ν/cm^-1^ (nujol): 2933, 2840, 1734, 1685, 1593, 1516.

*6-{N-[3-({3-[(E)-[(5-{[3,4,5-tris(pentyloxy)phenyl]carbonyloxy}pyridin-2-yl)methylidene]amino]- phenyl}methyl)phenyl]carboximidoyl}pyridin-3-yl 3,4,5-tris(pentyloxy)benzoate* **(4a):** From compound **20a** (79 mg, 0.16 mmol), 3,3’-methylenedianiline (16 mg, 0.08 mmol) and anhydrous EtOH (2 mL). This yielded a brown oil (52 mg, 56%); Calculated for C_69_H_88_N_4_O_10_ 73.12% C, 7.83% H, 4.94% N; found: 73.16% C, 7.87% H, 4.89% N; ^1^H-NMR (CDCl_3_): δ_H_ 8.60 (s, 2H, HC=N), 8.60 (d, 2H, Py-H, *J* = 2.6 Hz), 8.28 (d, 2H, Py-H, *J* = 8.6 Hz), 7.70 (dd, 2H, Py-H, *J* = 8.6, 2.6 Hz), 7.40 (s, 4H, Ar-H), 7.35 (t, 2H, Ar-H, *J* = 7.7 Hz), 7.16-7.13 (m, 6H, Ar-H), 4.07 (s, 2H, Ph_2_CH_2_), 4.07 (t, 4H, 2 x OCH_2_, *J* = 6.6 Hz), 4.04 (t, 8H, 4 x OCH_2_, *J* = 6.6 Hz), 1.88-1.68 (m, 12H, 6 x CH_2_), 1.52-1.31 (m, 24H, 12 x CH_2_), 0.92 (t, 18H, 6 x CH_3_, *J* = 6.99 Hz); ^13^C-NMR (CDCl_3_): δ_C_ 165.1, 161.3, 154.9, 153.7, 152.9, 150.6, 145.3, 145.1, 143.8, 131.8, 131.2, 129.2, 124.2, 123.8, 123.7, 120.5, 110.5, 75.4, 71.1, 43.6, 31.8, 30.7, 30.0, 24.3, 24.2, 15.8; MS m/z (ESMS): 1133 ([M]^+^, 100%); IR ν/cm^-1^ (nujol): 2927, 2854, 1736, 1587. 

*6-{N-[3-({3-[(E)-[(5-{[3,4,5-tris(heptyloxy)phenyl]carbonyloxy}pyridin-2-yl)methylidene]amino]- phenyl}methyl)phenyl]carboximidoyl}pyridin-3-yl 3,4,5-tris(heptyloxy)benzoate* (**4b**): From compound **20b** (287 mg, 0.51 mmol), 3,3’-methylene dianiline (50 mg, 0.25 mmol) and anhydrous EtOH (4 mL). This yielded a brown oil (195 mg, 59%); Calculated for C_81_H_112_N_4_O_10_ 74.73% C, 8.67% H, 4.30% N; found: 74.78% C, 8.70% H, 4.32% N; ^1^H-NMR (CDCl_3_): δ_H_ 8.60 (s, 2H, HC=N), 8.60 (d, 2H, Py-H, *J* = 2.6 Hz), 8.28 (d, 2H, Py-H, *J* = 8.6 Hz), 7.70 (dd, 2H, Py-H, *J* = 8.6, 2.6 Hz), 7.40 (s, 4H, Ar-H), 7.35 (t, 2H, Ar-H, *J* = 7.7 Hz), 7.16-7.13 (m, 6H, Ar-H), 4.07 (s, 2H, Ph_2_CH_2_), 4.07 (t, 4H, 2 x OCH_2_, *J* = 6.6 Hz), 4.04 (t, 8H, 4 x OCH_2_, *J* = 6.6 Hz), 1.88-1.71 (m, 12H, 6 x CH_2_), 1.52-1.30 (m, 48H, 24 x CH_2_), 0.88 (t, 18H, 6 x CH_3_, *J* = 7.0 Hz); ^13^C-NMR (CDCl_3_): δ_C_ 164.8, 159.9, 153.5, 152.4, 151.5, 149.2, 144.0, 143.8, 142.4, 130.5, 129.8, 127.9, 123.2, 122.8, 122.4, 119.1, 109.2, 74.0, 69.8, 42.2, 32.3, 32.2, 30.8, 29.7, 29.6, 29.4, 26.4, 23.0, 14.5; MS m/z (ESMS): 1324 ([M+Na]^+^), 1303; IR ν/cm^-1^ (nujol): 2927, 2854, 1739, 1586.

*6-{N-[3-({3-[(E)-[(5-{[3,4,5-tris(nonyloxy)phenyl]carbonyloxy}pyridin-2-yl)methylidene]amino]- phenyl}methyl)phenyl]carboximidoyl}pyridin-3-yl 3,4,5-tris(nonyloxy)benzoate* (**4c**): From compound **20c** (160 mg, 0.25 mmol), 3,3’-methylenedianiline (24 mg, 0.12 mmol) and anhydrous EtOH (4 mL). This yielded a white solid (117 mg, 65%); mp: 51 °C; Calculated for C_93_H_136_N_4_O_10_ 75.98% C, 9.32% H, 3.81% N; found: 75.90% C, 9.37% H, 3.85% N; ^1^H-NMR (CDCl_3_): δ_H_ 8.60 (s, 2H, CHN), 8.60 (d, 2H, Py-H, *J* = 2.2 Hz), 8.28 (d, 2H, Py-H, J = 8.6 Hz), 7.69 (dd, 2H, Py-H, *J* = 8.6, 2.2 Hz), 7.40 (s, 4H, Ar-H), 7.35 (t, 2H, Ar-H, *J* = 7.7 Hz), 7.16-7.13 (m, 6H, Ar-H), 4.06 (s, 2H, Ph_2_CH_2_), 4.06 (t, 4H, 2 x OCH_2_, *J* = 6.6 Hz), 4.04 (t, 8H, 4 x OCH_2_, *J* = 6.6 Hz), 1.88-1.71 (m, 12H, 6 x CH_2_), 1.48-1.27 (m, 72H, 36 x CH_2_), 0.88 (t, 6H, 2 x CH_3_, *J* = 7.0 Hz), 0.87 (t, 12H, 4 x CH_3_, *J* = 7.0 Hz); ^13^C-NMR (CDCl_3_): δ_C_ 163.6, 158.7, 152.3, 151.2, 150.3, 148.0, 142.8, 142.6, 141.3, 129.3, 128.6, 126.7, 122.0, 121.6, 121.2, 118.0, 108.0, 72.8, 68.6, 41.0, 31.1, 29.6, 28.9, 28.8, 28.6, 28.5, 25.3, 21.9, 13.3; MS m/z (ESMS): 1471 ([M+H]^+^); IR ν/cm^-1^ (nujol): 2927, 2854, 1738, 1587.

*6-{N-[3-({3-[(E)-[(5-{[3,4,5-tris(dodecyloxy)phenyl]carbonyloxy}pyridin-2-yl)methylidene]amino]- phenyl}methyl)phenyl]carboximidoyl}pyridin-3-yl 3,4,5-tris(dodecyloxy)benzoate* (**4d**): From compound **20d** (150 mg, 0.19 mmol), 3,3’-methylenedianiline (19 mg, 0.10 mmol) and anhydrous EtOH (4 mL). This yielded a white solid (110 mg, 67 %); mp: 55 °C; Calculated for C_111_H_172_N_4_O_10_ 77.40% C, 10.06% H, 3.25% N; found: 77.33% C, 10.01% H, 3.27% N; ^1^H-NMR (CDCl_3_): δ_H_ 8.60 (s, 2H, CHN), 8.60 (d, 2H, Py-H, *J* = 2.6 Hz), 8.29 (d, 2H, Py-H, *J* = 8.5 Hz), 7.69 (dd, 2H, Py-H, *J* = 8.5, 2.6 Hz), 7.40 (s, 4H, Ar-H), 7.35 (t, 2H, Ar-H, *J* = 7.7 Hz), 7.16-7.14 (m, 6H, Ar-H), 4.06 (s, 2H, Ph_2_CH_2_), 4.06 (t, 4H, 2 x OCH_2_, *J* = 6.6 Hz), 4.07 (t, 8H, 4 x OCH_2_, *J* = 6.6 Hz), 1.87-1.71 (m, 12H, 6 x CH_2_), 1.48-1.25 (m, 108H, 54 x CH_2_), 0.87 (t, 18H, 6 x CH_3_, *J* = 6.6 Hz); ^13^C-NMR (CDCl_3_): δ_C_ 164.3, 159.4, 153.0, 151.9, 151.0, 148.7, 143.5, 143.3, 142.0, 130.0, 129.3, 127.4, 122.7, 122.3, 122.0, 118.7, 108.7, 73.6, 69.3, 41.7, 31.9, 30.3, 29.6, 29.3, 26.0, 22.6, 14.0; MS m/z (ESMS): 1744 ([M+Na)]^+^); IR ν/cm^-1^ (nujol): 2927, 2854, 1735, 1585. 

*[Cu_2_(**3a**)_2_][PF_6_]_2_:* To a solution of compound **3a** (25 mg, 0.026 mmol) in propan-2-ol (5 mL) under a nitrogen atmosphere, [Cu(MeCN)_4_][PF_6_] (10 mg, 0.026 mmol) was added to give a dark red solution, which was heated under reflux overnight and then cooled to room temperature. A dark red solid precipitated from the solution on standing and was collected by filtration and washed with diethyl ether (41 mg, 67 %); Calculated for Cu_2_C_118_H_136_N_8_O_16_P_2_F_12_·H_2_O: 59.7% C, 5.9% H, 4.7% N; found: 59.4% C, 5.9%, 5.0% N; ^1^H-NMR (CD_2_Cl_2_): δ_H_ 9.04 (br, 4H, HC=N), 8.46 (br, 4H, Py-H), 8.25 (br, 4H, Py-H), 8.08 (d, 4H, Ar-H, *J* = 8.0 Hz), 7.79 (d, 4H, Py-H, *J* = 8.6 Hz), 7.60 (s, 4H, Ar-H), 7.27 (t br, 4 H, Ar-H, *J* = 8.6 Hz), 7.00 (s br, 12H, Ar-H), 6.96 (d, 4H, Ar-H, *J* = 8.5 Hz), 4.09 (t, 8H, 4 x OCH_2_, *J* = 6.5 Hz), 4.04 (t, 8H, 4 x OCH_2_, *J* = 6.5 Hz), 3.69 (br s, 4H, Ph_2_CH_2_), 1.88-1.68 (m, 16H, 8 x CH_2_), 1.52-1.31 (m, 32H, 16 x CH_2_), 0.96 (q, 24H, 12 x CH_3_, *J* = 7.0 Hz); m/z (+ve FABMS): 2194 [(Cu_2_(3**a**)_2_)(PF_6_)]^+^; MS m/z (ESMS): 1024 [(Cu_2_(**3a**)_2_)]^2+^; IR ν/cm^-1^ (solid): 2953, 2926, 2867, 1727, 1594, 1556, 1521; UV/Vis (CH_2_Cl_2_) λ_max_ (ε, L mol^-1^ cm^-1^): 495 (9,500), 312 (83,600) nm.

*[Cu_2_(**3b**)_2_][PF_6_]_2_:* The same procedure as described for the preparation of compound [Cu_2_(**3a**)_2_][PF_6_]_2_ was followed, using compound **3b** (12 mg, 0.011 mmol) and [Cu(MeCN)_4_][PF_6_] (4 mg, 0.011 mmol). This yielded a dark red solid (20 mg, 70 %); ^1^H-NMR (CD_2_Cl_2_): δ_H_ 9.03 (br s, 4H, HC=N), 8.53 (br s, 4H, Py-H), 8.23 (d, 4H, Py-H, *J* = 8.3 Hz), 8.08 (d, *J* = 4H, Ar-H, 8.3 Hz), 7.77 (d, 4H, Py-H, *J* = 8.5 Hz), 7.59 (s, 4H, Ar-H), 7.25 (br t, 4H, *J* = 7.5 Hz), 7.00 (s br, 12H, Ar-H), 6.98 (br s, 4H, Ar-H), 4.08 (t, 8H, 4 x OCH_2_, *J* = 6.5 Hz), 4.02 (t, 8H, 4 x OCH_2_, *J* = 6.5 Hz), 3.66 (br s, 4H, Ph_2_CH_2_), 1.88-1.71 (m, 16H, 4 x CH_2_), 1.52-1.30 (m, 64H, 32 x CH_2_), 0.90 (m, 24H, 12 x CH_3_); m/z (+ve FAB MS): 2419 [Cu_2_(**3b**)_2_(PF_6_)]^+^, 1137 [Cu(**3b**)]^+^; MS m/z (ESI MS): [Cu_2_(**3b**)_2_]^2+^ ; IR ν/cm^-1^ (solid): 2922, 2852, 1727, 1595, 1556, 1513; UV/Vis (CH_2_Cl_2_) λ_max_ (ε, L mol^-1^ cm^-1^): 480 (11,400), 294 (94,600) nm.

*[Cu_2_(**3c**)_2_][PF_6_]_2_:* The same procedure as described for the preparation of compound [Cu_2_(**3a**)_2_][PF_6_]_2_ was followed_,_ using compound **3c** (24 mg, 0.018 mmol) and [Cu(MeCN)_4_][PF_6_] (7 mg, 0.018 mmol). This yielded a dark red solid (56 mg, 65 %); The ^1^H-NMR spectrum of the red material was very broad and the sample did not ionise well in ESI-MS; IR ν/cm^-1^ (solid): 2919, 2853, 1732, 1662, 1600, 1508; UV/Vis (CH_2_Cl_2_) λ_max_ (ε, L mol^-1^ cm^-1^): 482 (7 400), 298 (94 700) nm.

## 4. Conclusions

In summary, we have successfully synthesised a series of novel banana-shaped compounds, in which the bend unit is formed by a 4,4’-methylenedianiline or 3,3’-methylenedianiline core bearing two symmetric pyridylimine linkages. Also, a number of complexes [Cu_2_(**3a-c**)_2_][PF_6_]_2_ were formed by coordination with Cu(I) cations. Mass spectrometry techniques have shown that dinuclear double stranded arrays were formed. Thermal analyses of the ligands **1a-d**, **2a-d**, **3a-c** and **4a-d** and the metal complexes [Cu_2_(**3a-c**)_2_][PF_6_]_2_ revealed that these do not display any mesophase behavior. However, the generic structure of the target series **1a-d**, **2a-d**, **3a-c** and **4a-d** appears to fulfill, in principle, the same criteria of the banana-shaped mesogens known to date [37]. Further work is being carried out to prepare and investigate the liquid crystalline properties of the Cu (I) complexes formed from ligands **1a-d**, **2a-d** and **4a-d**, because such metal containing structures conjure up potential application of magnetic LC phases.
